# The Impact of COVID-19 on HIV Treatment and Research: A Call to Action

**DOI:** 10.3390/ijerph17124548

**Published:** 2020-06-24

**Authors:** Tiffany Chenneville, Kemesha Gabbidon, Patricia Hanson, Cashea Holyfield

**Affiliations:** 1 Department of Psychology, University of South Florida St. Petersburg, 140 7th Avenue South, St. Petersburg, FL 33701, USA; kgabbido@usf.edu; 2 Department of Psychological and Social Foundations, University of South Florida, 4202 E. Fowler Avenue, Tampa, FL 33620, USA; yaa1@usf.edu (P.H.); cholyfield@usf.edu (C.H.)

**Keywords:** COVID-19, coronavirus, HIV, AIDS, health disparities

## Abstract

The impact of the COVID-19 pandemic is far reaching, with devastating effects on individuals, communities, and societies across the world. People with chronic health conditions may be at greater risk of contracting or experiencing complications from COVID-19. In addition to illness or death for those who contract the virus, the physical distancing required to flatten the curve of new cases is having a negative impact on the economy, the effects of which intersect with mental health and other existing health concerns, thus affecting marginalized communities. Given that HIV also has a disproportionate impact on marginalized communities, COVID-19 is affecting people with HIV (PWH) in unique ways and will continue to have an impact on HIV research and treatment after the COVID-19 crisis passes. Using the biopsychosocial framework to contextualize the impact of COVID-19 on PWH, the purpose of this review article is to: (1) outline the similarities and differences between the COVID-19 and HIV pandemics; (2) describe the current and future impact of COVID-19 on PWH; and (3) outline a call to action for scientists and practitioners to respond to the impact of COVID-19 on HIV prevention and treatment.

## 1. Introduction

The first cases of COVID-19 appeared in Wuhan, China in December 2019 [1]. Similar to other severe acute respiratory viruses (e.g., SARS-CoV), COVID-19 is a highly contagious disease but with an even higher reproduction number, which refers to the average number of people a person with the virus will infect, thus serving as a metric of how easily a virus is transmitted [2]. COVID-19 has spread quickly throughout the world. COVID-19 results in mild illness for most people who contract the virus [3]. However, people who are older in age or have pre-existing conditions are at risk of severe illness or death [3]. Healthcare systems in many countries are taxed, particularly in areas where infection rates are high, resulting in health implications not only for patients with COVID-19 but also for other people who require healthcare services. The WHO’s [3] recommendation for physical distancing to limit the spread of COVID-19, echoed by the Centers for Disease Control and Prevention [4] in the United States (US), has resulted in stay-at-home orders in many places. The economic and psychosocial toll of self-quarantining is significant as people face challenges associated with unemployment or underemployment as well as anxiety, fear, and grief, not to mention the deleterious effects of social isolation. In addition, many families are confronted with the added burden and stress of juggling the virtual educational demands of school-aged children due to school closures.

People with HIV (PWH) are particularly vulnerable during the time of COVID-19. Although recent research suggests that PWH may not be contracting COVID-19 at disproportionate rates, which is hypothesized to be a function of antiretroviral treatment (ART; [5]), PWH who are not taking ART or whose disease is not well managed may be at increased risk for contracting COVID-19 due to having a compromised immune system and also may be at increased risk for serious symptoms and death. Beyond their increased risk for complications resulting from COVID-19, PWH are affected by the COVID-19 crisis in a myriad of other ways. For example, PWH are likely to experience treatment interruptions due to restrictions on non-emergency medical appointments related to physical distancing requirements. Although many healthcare providers are offering telehealth services, the transition to telehealth has been slow in some places, and the uptake of telehealth services is variable. Indeed, some PWH may not have the resources needed to fully take advantage of telehealth opportunities (e.g., access to devices or adequate Internet service). As healthcare facilities re-open and resume normal daily operations, PWH face the risk of exposure to COVID-19 when attending medical appointments. Given the high rates of mental health issues (e.g., anxiety and depression) that exist among this population, PWH are particularly vulnerable to the effects of isolation resulting from restricted mobility associated with the pandemic. Furthermore, to the extent that COVID-19 is affecting marginalized communities in unique ways, it also is affecting PWH in unique ways, given that HIV disproportionately affects people from marginalized groups. Indeed, the pandemic’s disproportionate impact on people of color and those who are economically disadvantaged is making apparent existing health disparities that also exist within the HIV epidemic. As another example of the impact of COVID-19 on marginalized communities, Lodgell and Kuchukhidze [6] described the impact of COVID-19 on migrant workers who are living with HIV.

Scientists and practitioners are in a position to respond to the needs of PWH in the time of COVID-19. Using the biopsychosocial framework to contextualize the impact of COVID-19 on PWH, the purpose of this article is threefold: (1) to outline the similarities and differences between the COVID-19 and HIV pandemics; (2) to describe the current and future impact of COVID-19 on PWH including the short-term and long-term effects of the current crisis on HIV research and treatment; and (3) to outline a call to action for scientists and practitioners to respond to the impact of COVID-19 on PWH and HIV prevention efforts.

## 2. Biopsychosocial Framework

Taking into account the connection between mind and body, the biopsychosocial model acknowledges the impact of interacting biological, psychological, and social factors on health and health outcomes [7,8]. The biopsychosocial framework has been used to better understand a variety of diseases and medical conditions, such as postpartum depression [9], multiple sclerosis [10], and spinal cord injuries [11] as well as HIV. For example, recent research has incorporated the biopsychosocial framework into HIV research on stigma among older adults [12], pain and substance abuse [13], and fatigue [14], to name just a few studies. The biopsychosocial model also recently was used by an international panel of experts to guide practice for pain management during the COVID-19 pandemic [15]. Thus, the biopsychosocial framework is well suited to contextualize a discussion of the similarities and differences between COVID-19 and HIV and the impact of COVID-19 on HIV treatment and research as well as scientists’ and practitioners’ potential response.

## 3. Similarities and Differences between COVID-19 and HIV

### 3.1. Brief Overview and Modes of Transmission

HIV is a member of the genus Lentivirus and is part of the family Retroviridae [16]. Lentiviruses are slow moving, characterized by long incubation periods and illness duration. HIV acts primarily by depleting the immune system cells, namely macrophages and CD4+ cells, which leaves one vulnerable to opportunistic infections. Globally, the HIV-1 subtype is responsible for most infections, whereas the HIV-2 subtype is most prevalent in West Africa [16]. HIV transmission occurs through exposure to infected bodily fluids (e.g., blood, semen, vaginal fluids, breast milk). The most common transmission routes are through condomless sexual intercourse, intravenous drug use and occupational exposures, and from mother-to-child during pregnancy, delivery, or breastfeeding.

Comparatively, severe acute respiratory syndrome coronavirus 2 (SARS-CoV-2), also known as the 2019 novel coronavirus (2019-nCoV), is responsible for the COVID-19 pandemic. Unlike HIV, SARS-CoV-2 is an acute respiratory infection with a short incubation period. SARS-CoV-2 is a positive-sense single stranded RNA virus, genetically similar to SARS-CoV [17], seen in the 2003 global pandemic. The novel SARS-CoV-2 belongs to the genus β-CoVs (Betacoronavirus) and the subgenus Sarbecovirus [17]. Most recent evidence shows respiratory droplets and contact as the primary routes of transmission [17]. Preliminary research also showed the potential for oral-fecal transmission [18,19]. Concurrent research provides some evidence that the virus is viable on plastic and steel surfaces but less viable on cardboard or copper [20]. The duration of SARS-CoV-2′s viral shedding or period of infectivity remains unknown; however, the incubation period is believed to be similar to that of other coronaviruses, which is 2–14 days. With HIV, an undetectable viral load means the virus is un-transmissible [21], but this is not the case with SARS-CoV-2, as presence of the viral RNA does not indicate a current infection [22]. 

### 3.2. Reproductive Ratio

As briefly mentioned, the basic reproduction number (R_0_) is defined as the expected number of secondary cases when one case interacts with a susceptible population [23]. Susceptible populations are those without any acquired immunity either through previous exposure or immunization. As described by Hsieh and Wang [23], R_0_ has been used to determine the capacity for emerging infectious diseases to become endemic.

R_0_ values are highly susceptible to interventions (e.g., medication use, drug resistance, behavior modification). An example of this was demonstrated by Velasco-Hernandez and colleagues [24], who assessed the R_0_ value for HIV among men who have sex with men (MSM) in San Francisco, in which 50–90% of the cases received antiretroviral therapy (ART). The authors determined an R_0_ of 0.90 if risky sexual behavior was reduced and approximately 10% of the treated cases developed resistance to ART. However, if no change occurred in sexual behaviors and 10% of the treated cases developed resistance to ART, the R_0_ was estimated to increase to 1.0 [24]. Finally, if risky sexual behavior increased and 10–60% of the population developed ART resistance, the R_0_ then increased to 1.16 [24]. Overall, HIV has a relatively low R_0_ value when ART is used.

Comparatively, the R_0_ value of SARS-CoV-2 is quite fluid. Liu and colleagues [2] reported a mean R_0_ of 3.28. This is similar to the 2003 SARS-CoV pandemic [25]. However, it should be noted that the methodology used affects the R_0_ value [2]. For example, mathematical models often show higher R_0_ values than when stochastic and statistical methods are used [2]. For context, it is important to compare SARS-CoV-2 to other respiratory infections. In a systematic review conducted to determine R values for pandemic and seasonal influenza infections, Biggerstaff and colleagues [26] identified the following mean R_0_ values: 1.80 (1918 H1N1 pandemic); 1.65 (1957 H2N2 pandemic); 1.80 (1968 H3N2 pandemic); 1.46 (2009 SARS-CoV pandemic) and 1.28 (seasonal influenza). Across these viral infections, both coronaviruses demonstrate higher rates of transmissibility than influenza and HIV.

### 3.3. Risk Factors

It is important to think about both *who* and *how* when considering risk factors for disease. For COVID-19, failure to adhere to physical distancing or hand washing recommendations constitute behaviors that increase risk for contracting COVID-19 (i.e., the *how*). The *who* include people who are older in age or who have pre-existing conditions such as chronic respiratory disease, cardiovascular disease, hypertension, cancer and diabetes when describing risk factors for the coronavirus [27,28]. Comparatively, when describing risk factors for HIV, “risky” sexual or drug use behaviors are most commonly associated with the *how* of transmission, although HIV can also be transmitted from mother-to-child or through other means of exposure to blood (e.g., tainted blood transfusions). In terms of the *who*, it is well established that HIV disproportionately affects groups already marginalized, including racial/ethnic and sexual or gender minorities, as well as those living in poverty. It is quickly being recognized that COVID-19 also disproportionately affects minority groups [29], which is not surprising given existing health disparities as described in more detail below.

### 3.4. Impact on Economy

Although the impact of the COVID-19 pandemic on economies across the world [30] including developed nations such as the US [31] is not yet entirely known, there is no question that COVID-19 is having an impact on the global economy [32,33]. A summary of research in this area suggested that a recession is likely [34]. Maital and Barzani [34] explained that COVID-19 is having its greatest impact on the supply side of the economy, yet most solutions are being offered or implemented on the demand side given that little is known about how to effectively address the economic impact on the supply side. Clearly, the immediate economic impact of COVID-19 is more severe and far reaching than the economic impact of HIV. However, there is an economic impact to HIV prevention and treatment. The average lifetime cost of HIV treatment has been estimated at the US equivalent of USD 441,708 (based on the value of the US dollar in 2020; [35]). There are also costs associated with the treatment of HIV-related illnesses or mental health conditions as well as HIV prevention efforts. Vaccine research is underway for COVID-19 and HIV; however, a COVID-19 vaccine is likely to be developed long before a vaccine is developed for HIV. Thus, while the immediate impact of COVID-19 on the economy is far greater, the economic impact of HIV will continue long after the COVID-19 pandemic is resolved.

### 3.5. Psychosocial Implications

Despite differences in how the viruses are spread, HIV and COVID-19 have in common fear and anxiety related to transmission. Similar to the early days of the HIV epidemic, when information about treatment and prevention was lacking, there is a lot of fear about contracting COVID-19 in the absence of a vaccine or scientifically proven treatments to address symptoms and prevent death. Such fear and anxiety have implications for mental health and promotes disease-related stigma. HIV-related stigma is well established and, because of its impact on HIV testing and treatment, research on effective HIV-stigma reduction interventions is prevalent (see Stangl and colleagues [36] for a systematic review of the literature). There is emerging evidence of the mental health burden of pandemic fear related to COVID-19 [37] and its potential impact on preventive behavior [38]. There is also emerging evidence of stigma and discrimination related to COVID-19 [39].

Mental health issues such as anxiety, depression, and posttraumatic stress and substance use are common among PWH with mental health issues and are considered both a cause and a consequence of HIV. Similarly, as Ho and colleagues [40] noted, pandemic fear in general can worsen existing mental health disorders and also may result in new diagnoses. For COVID-19 specifically, social isolation is likely to contribute to mental health issues, especially among the elderly [41,42]. Acknowledging the mental health impact of COVID-19, WHO [43] published recommendations for addressing mental health and psychosocial issues during the COVID-19 outbreak.

### 3.6. Health Disparities

As with HIV, the COVID-19 pandemic reveals the systemic inadequacies that produce health disparities. What these illnesses demonstrate is a disproportionate burden on already vulnerable populations experiencing poverty and other systemic stressors. The United Nations’ [44] report has indicated that increases in food costs and market stockpiling has had the most harmful impact on vulnerable communities, particularly those in low income nations. Although vulnerable populations vary across nations, those with stigmatized or marginalized intersecting identities often experience the highest burden, including MSM, transgender women, people who inject drugs, commercial sex workers, young women, and youths (15–24), who account for a third of all new HIV infections [45]. Furthermore, immigrants are at increased risk of infectious diseases, including both HIV and COVID-19 [6], as are other people who are displaced.

Not surprisingly, the COVID-19 pandemic also appears to more commonly affect those with marginalized intersecting identities, exacerbating racial/ethnic, socioeconomic, disability status, and age-related disparities. This is demonstrated in the disease risk and case fatality rates. In the American context, for example, approximately 50% of COVID-19 cases and 70% of deaths in Chicago occurred among African American/Black males, who only account for 30% of the city’s population [29]. Similar disparities can be seen in New York City, Louisiana, Michigan, and other regions of the US [46,47,48]. Furthermore, comparable disparities among Hispanics are beginning to emerge; as of late April 2020, Hispanics experienced the highest COVID-19 case fatality rate in New York City [47].

### 3.7. Government Response

On 31 March 2020, the UN [44] called for a comprehensive and coordinated global response to the COVID-19 pandemic. In addition to supporting short-term measures such as limiting access to critical resources and creating travel bans, the UN [44] urged global political leaders to develop a sustained coordinated response. Unfortunately, this has not yet happened in all countries. At the time of writing, the US was the viral epicenter, yet had struggled to enforce an organized national response, instead relying heavily on state and local measures that vary considerably [49]. The most immediate response from the majority of nations (166 countries as of March 2020) was to close schools and universities [50]. However, other actions varied by country. For example, the US Food and Drug Administration used Emergency Use Authorization to expedite approval of potential medical interventions and waived enforcement protocols to allow non-health care industries to develop technological advances to challenge the pandemic [50]. Likewise, the Centers for Medicare and Medicaid Services in the US opted to facilitate an expansion of patient care sites [50].

Globally, the health care response has focused on building the workforce capacity. This includes (a) increasing the number of health care professionals available by bringing in medical professionals from other nations or adjusting medical license requirements, (b) using technology to reduce provider burden, and (c) collaborating with non-health care partners to address shortages of personal protective equipment [50]. On the economic level, governments in wealthy nations have established emergency funds to provide economic reprieve. For example, the US expanded unemployment qualifications and provided economic stimuli to some, and other nations such as South Korea and Canada developed emergency funds [50]. Some nations opted to subsidize wages (New Zealand, Australia, Canada, and United Kingdom; [50]. Economically disadvantaged nations remain vulnerable and their health care infrastructure stands to be further weakened as they address this pandemic.

The global response to HIV epitomizes the convergence of politics and public health care. Early on, the response was largely characterized by robust grassroot and global political efforts. Grassroots efforts among those most affected by HIV would serve as the initial global response to the pandemic. Many governments were criticized for their slow, moralistic response. It was activists and non-profit/non-governmental organizations that organized to advocate for immediate government response and to provide education and support to the affected local communities [51]. However, the government response has played a complicated role in the history of HIV/AIDS. Successes include an international agenda focused on HIV; the development of UNAIDS; and the creation of the Global Fund to Fight HIV, Tuberculosis, and Malaria, among other efforts, resulting in billions in funding [52]. However, governments—or rather, their policies—have played a significant role in prohibiting progress, including laws that criminalize the behaviors of PWH, same-sex relationships, and transgender individuals; a restriction of services for key populations; limited access to ARTs for low-to-middle income nations; failure to provide comprehensive sex education within school settings; and poor support for evidence-based harm reduction programs [53,54]. Additionally, global financial support for HIV has declined steadily over the past few years, with many nations now viewing HIV as a non-priority [53].

## 4. Short-Term and Long-Term Impact of COVID-19 on HIV Treatment

COVID-19 has significantly altered everyday life for individuals worldwide. The presence of the virus has introduced physical distancing and closed schools and businesses, resulting in major disruptions to daily functioning. Given the social nature of human beings, people are finding ways to adjust to COVID-19 for the foreseeable future. In addition to the short-term effects of COVID-19, it is also critical for health care providers (e.g., doctors, psychologists, social workers, case managers, etc.) to consider how the impacts of COVID-19 may affect PWH, and the provision of health care treatment across time. For PWH, adjusting to COVID-19 may have significant effects on the biological, psychological and social aspects of their lives.

### 4.1. Biological Impact of COVID-19 on HIV Treatment

PWH whose disease is not well managed are placed at an increased risk for contracting and experiencing complications related to COVID-19, in addition to complications related to HIV disease progression. Given the lifelong prognosis of HIV, it is imperative for PWH to regularly visit their healthcare providers and adhere to treatment [55]. PWH’s treatment may be interrupted or otherwise affected as a result of stay at home orders. For example, in America, many health care providers canceled in-person appointments and transitioned to telehealth appointments in order to comply with federal guidelines [56]. However, telehealth services are limited in the range of services that can be provided to clients [57], thus, PWH may be unable to fully access the services required for their HIV treatment. Additionally, some PWH may be unable to access telehealth services for various reasons (e.g., lack of access to technology, limited knowledge of telehealth, etc.), which can hinder their treatment progression.

PWH are also more likely to contract opportunistic infections (e.g., pneumonia, tuberculosis, toxoplasmosis, etc.; [58]), than those without compromised immune systems. PWH who are experiencing any additional illnesses may experience delayed treatment due to COVID-19. This can occur due to hospital overcrowding in an already taxed healthcare system. PWH who seek out urgent care may face an increased risk of contracting COVID-19 among other illnesses while in healthcare settings [59].

### 4.2. Psychological Impact of COVID-19 on HIV Treatment

Given the unprecedented nature of the COVID-19 outbreak, an increase in anxiety has been prevalent worldwide. Furthermore, the CDC [60] has noted that individuals with chronic health conditions, such as HIV, may develop a stronger stress response than the rest of the population. This strong stress response is due to an increased risk of contracting COVID-19 due to a compromised immune system.

Stress during an outbreak can lead to maladaptive coping mechanisms, which may include increased alcohol intake. PWH who drink one or more alcoholic beverages a day are at greater risk of death and/or other alcohol-related health issues than individuals without HIV [61]. Alcohol consumption reduces immune system strength [61], thus alcohol consumption can weaken a PWH’s ability to fight HIV and COVID-19. Additionally, WHO [62] reported that alcohol consumption can increase one’s risk for health problems. Alcohol consumption for PWH also increases viral load over time [63,64]. Alcohol can lead to impaired judgment, such as engaging in risky sexual behaviors [61], 2017). Risky behaviors paired with an increased viral load may result in PWH passing on the virus through condomless sex. Although people are practicing physical distancing, individuals who are quarantined with a romantic partner may be engaging in sexual activities more often [65], which may be problematic for PHW whose viral load is not well managed and who do not engage in protective behaviors (e.g., condom use) or whose sexual partners are not using pre-exposure prophylaxis (PrEP). Additionally, the use of alcohol may exacerbate symptoms of depression and anxiety [66].

PWH are two to four times more likely to develop depression than those without the virus [67,68,69]. Depression is also the most common mental health disorder among PWH (40–42%; [70]. Thus, the physical distancing required to combat COVID-19 may increase loneliness, which can in turn exacerbate depressive symptoms. Furthermore, because HIV has a higher prevalence in populations experiencing poverty [71], PWH may be limited in accessing resources to cope with physical distancing (e.g., cell phones, laptops, internet service). Depressive symptoms that do not necessarily meet diagnostic criteria for a depressive disorder have also historically been linked to worse health outcomes for PWH, including impaired immunological response and mortality [72,73]. Though the relationship between depression and treatment adherence among PWH may not be causal, research has found that depressive symptoms such as loss of interest, feelings of worthlessness, and thoughts of death or suicide may have negative effects on PWH’s desire to take their medications, or engage in the necessary medication management activities that are conducive to a healthy lifestyle [74,75,76]. For this reason, health care providers who work with PWH who are depressed may have to work even harder following COVID-19 to ensure treatment adherence and participation in therapy activities.

Given the importance of mental health and continuation of services, many mental health providers have had to provide services to patients via telehealth. Thus, it is possible that in the future an increased number of providers may opt to continue providing services via telehealth in order to increase attendance rates for regularly scheduled appointments, and expand access to care by cutting patient costs associated with travel, parking, childcare, and time off work [77]. Despite the potential benefits of telehealth, these services are not met without several potential limitations including concerns related to client confidentiality, difficulty with internet connections which may affect audio and visual quality during sessions, and lack of face to face contact which can affect the establishment of rapport and the therapeutic alliance between a client and psychologist or health care provider.

Furthermore, due to the potential impacts of psychological conditions (e.g., anxiety, depression, PTSD), COVID-19, and health-related concerns among PWH, there may be an increase in the number of PWH who seek mental health services following the COVID-19 pandemic. It is important to note, however, that the mental health effects of COVID-19 may not be apparent for every individual immediately following the pandemic. For this reason, it is also possible that in the coming years, there will be an even greater need for health and mental health care providers to support the economic and psychological impacts of COVID-19. Preventing, detecting, and responding to mental health conditions should be an important component of short- and long-term global health efforts.

### 4.3. Social Impact of COVID-19 on HIV Treatment

As COVID-19 continues to spread worldwide, most international health bodies have introduced guidelines about physical proximity, which often include remaining two meters (six feet) away from one another in public settings, although some governments have recommended that one meter is sufficient in fast-moving public settings, since time as well as distance appears to affect exposure. As an example of guidelines in the American context, the CDC recommended limiting one’s interactions with anyone outside of their household and limiting one’s trips to only those that were essential (e.g., working an essential job or going to the grocery store) early in the pandemic [60]. Although many countries issued mandatory stay-at-home orders, it is important to note that guidelines have varied and are changing from country to country.

Although physical distancing guidelines serve to benefit one’s physical health, these guidelines may be detrimental to one’s social and emotional health. PWH may be even more reluctant to engage in physical interaction with others. This may be especially true for newly diagnosed PWH who do not have a full understanding of the ways in which HIV is transmitted.

PWH who do not have the means to connect with their friends digitally may also struggle to maintain those social bonds. PWH, like others, may be spending more time on social media sites in order to connect with others. Bekalu and colleagues [78] found that using social media for emotional connection can decrease one’s social well-being, positive mental health and self-rated health.

Because many PWH are part of marginalized communities, other social impacts also exist. For example, increased risk exists for PWH whose domestic arrangements may be risky or violent; who have reduced access to drugs normally bought on the street, or reduced access to needle exchange schemes, which are important for secondary HIV prevention; who are unemployed or underemployed or who have been furloughed as a result of COVID-19; who are homeless or have unstable housing as a pre-existing situation or as a result of COVID-19; who may need to resort to food banks and other community resources in order to survive during the pandemic; who may have reduced access to medical or pharmacy services as a result of reduced public transport services; or who are imprisoned or in other institutional care. There are also social impacts on people in rural and remote areas where internet services are limited or weather-dependent, or whose resources require that they subscribe to limited data plans. Furthermore, PWH who were separated from partners or family when international borders closed and who are now facing long separations from their primary social supports are negatively affected by COVID-19 in unique ways. Finally, there are socio-political impacts related to COVID-19 for PWH. Although the political response to COVID-19 has been good in many countries, responses have been marked by controversy in other countries. Confused or delayed responses resulting from the absence of consistent public messaging in some countries has affected access to COVID-19-related services and resources for PWH. Clearly, the social impact of COVID-19 on HIV treatment is far-reaching and multifarious, and the long-term impact is, in many ways, unforeseeable.

## 5. Short-Term and Long-Term Impact of COVID-19 on HIV Research

In response to the COVID-19 pandemic, research and academic institutions have halted or modified their research activities. In the short term, this results in delays in research progress, a need to transition to remote data collection methods when applicable, and redirecting research efforts to address the COVID-19 crisis [79]. Essentially, researchers will lose progress in the battle against HIV and other chronic illnesses such as heart disease, cancer, and diabetes. To better understand the impact on HIV research, below are examples of disruptions to HIV-related biological, psychological, and social research. While some short-term adjustments are possible, long-term implications are far more difficult to predict. However, many researchers anticipate that future funding to non-COVID-19 research will see reductions [80]. More specifically, there is an expectation of declines in philanthropic and governmental support and research/grant funding [81].

### 5.1. Impact on Biomedical HIV Research

Biomedical research includes the investigation of the biological process and the causes of disease. It includes basic, applied, and clinical research. HIV-related biomedical research may include investigating the molecular mechanism underlying HIV-associated neurocognitive disorder, developing cost-effective prevention technologies, or determining the interaction of HIV with non-communicable diseases. Across the globe, some biomedical researchers have opted to continue long-term experiments, maintain vital equipment, cell lines, and other time-sensitive research items with the use of a skeleton crew (least number of research team members needed; [82]. Other experiments have been discarded or frozen for use later as they wait for directives on next steps [82].

### 5.2. Impact on Psychological HIV Research

Socio-behavioral and psychological HIV research currently remains chronically underfunded and may continue to experience additional declines in funding following the pandemic. Examples of HIV-related socio-behavioral and psychological research includes HIV-stigma reduction interventions, cognitive and behavioral health interventions, intersectionality and HIV prevention, treatment, and care. Depending on the study methodology, these research studies can be modified to be implemented virtually; however, careful considerations must be given to research ethics (e.g., privacy and confidentiality). Other studies that require lab equipment and space will need to be delayed and implemented later. Researchers may choose to modify their research studies; for example, psychological researchers may choose to investigate the acceptability and uptake of HIV home testing kits and telehealth counseling services. In addition, project deliverables will need to be modified as recruitment and enrollment may be negatively impacted by the COVID-19 pandemic.

### 5.3. Impact on Social Factors of HIV Research

Much like psychological research, social science research remains underfunded and undervalued and may also experience declines in funding following the pandemic. When feasible, researchers may need to think creatively about redesigning their studies. For example, HIV researchers may be interested in assessing and implementing interventions to mitigate the impact of COVID-19 on vulnerable communities, which may include assessing the impact of COVID-19 on HIV prevention, treatment, and care efforts, or strengthening COVID-19 diagnostic and care capacity for PWH. When considering social science research, aspects of participatory research will experience severe challenges, particularly community-engaged research. Physical distancing requirements prevent the use of in-person focus groups and interviews. In addition, research that requires partnership development with gatekeepers often requires time and resource-intensive efforts and may not be effectively transitioned to virtual or other remote settings. Besides the logistical difficulties to host and plan community advisory meetings, train facilitators, host focus groups, and implement interventions, the psychological toll of the pandemic would likely undermine recruitment and enrollment efforts.

## 6. Call for Action for Scientists and Practitioners

Scientists and practitioners are in a unique position to respond to the impact that COVID-19 is having on PWH and HIV prevention efforts, including research. To address HIV treatment needs now and in the future, providers working with PWH are encouraged to obtain training in telehealth. The American Psychological Association’s (APA) Joint Task Force for the Development of Telepsychology Guidelines for Psychologists [83] may be particularly helpful for mental health care providers (even those not residing in the US) who are transitioning to telehealth or hoping to strengthen their knowledge and skills in this area. Scientists conducting HIV research are encouraged to be creative and flexible when transitioning current studies into virtual formats (when doing so is feasible) and designing future HIV studies. With regard to the latter, researchers may want to consider the ways in which HIV projects may be responsive to COVID-19 or other pandemic funding opportunities. For example, it might be possible to secure funding to add a COVID-19-specific aim to an existing externally funded HIV project, or to incorporate HIV-related specific aims to new COVID-19 projects. To these ends, interdisciplinary collaborations may be useful. Finally, scientists and practitioners are encouraged to continue to contribute to efforts to reduce health disparities. This will require expanding economic and social support, building trust and social cohesion, denouncing hate, and collecting more and better data [84].

## 7. Conclusions

This paper outlined similarities and differences between the COVID-19 and HIV pandemics, described current and future impacts of COVID-19 on HIV treatment and research using a biopsychosocial framework, and delineated a call to action for scientists and practitioners by stimulating readers to consider how this framework applies to their local situation. Pandemics have the capacity to illustrate the role of social, political, and economic contexts in the emergence and management of illnesses in our globalized community. The informal slogan, “we are in this together,” will continue to ring true of our responses to current and future pandemics like HIV and COVID-19. Health and mental health care providers are urged to advocate for systemic changes that lessen the inequity experienced by marginalized and disadvantaged communities. In addition, it is important for governments to recognize that the economic cost of infectious disease outbreak management will remain more costly than that of health promotion and disease prevention [85]. A commitment to health promotion and disease prevention would lessen the frequency and severity of emerging infectious diseases and would ensure that critical progress and advances in biomedical and social-behavioral research are not negatively impacted. It is essential to recognize that all pandemics have biological, psychological, and social implications, of which health care providers play a crucial role in the preparedness and global response.
